# Artistic anti-counterfeiting with a pH-responsive fluorescent ink using DFT and molecular electrostatic potential mapping insights

**DOI:** 10.1038/s41598-025-03982-w

**Published:** 2025-06-02

**Authors:** Hebat-Allah S. Tohamy

**Affiliations:** Cellulose and Paper Department, National Research Centre, 33 El Bohouth Str., P.O. 12622, Dokki, Giza Egypt

**Keywords:** Fluorescent ink, DFT, pH-sensitive ink, Anti-counterfeiting, Artistic ink, Security ink, Onion peels, Chemistry, Materials science, Nanoscience and technology, Optics and photonics

## Abstract

The observed fluorescence behavior of the sulfur, nitrogen-doped carbon dots (S, N-CDs) ink which derived from onion peel wastes (OW) demonstrates its pH-sensitive nature, making it suitable for applications where visual or fluorescent changes upon pH variation are desired. The initial lack of fluorescence under UV light suggests that the S, N-CDs in the ink are in a non-fluorescent state. However, upon treatment with acid, the ink exhibits a faint yellow color under light and fluoresces under UV light. This indicates a shift in the electronic structure of the S, N-CDs, likely due to protonation. The return to non-fluorescence after re-treatment with alkaline solution suggests that the de-protonation process reverses the effect of acid, restoring the S, N-CDs to their original non-fluorescent state. This reversible pH-sensitivity is a valuable asset for various applications. The synthesized S, N-CDs exhibited a reversible change in fluorescence intensity under acidic and alkaline conditions, transitioning from non-fluorescent to fluorescent under acidic conditions and back to non-fluorescent in alkaline media. Density Functional Theory (DFT) calculations revealed that S, N-doping resulted in a narrower energy gap (0.2779 eV compared to 0.3199 eV for N-CDs) and a higher dipole moment (2.640 Debye), enhancing their reactivity towards protons and leading to more pronounced color and fluorescence changes across different pH conditions. The S, N-CDs displayed dual fluorescence emission peaks at 443.00 nm and 502.00 nm upon excitation at 350 nm, and fluorescence contour maps (FCM) confirmed their multicolor emission capabilities. The calculated quantum yield for the S, N-CDs was notably high at 37.76%. Fourier Transform Infrared (FTIR) spectroscopy confirmed the successful incorporation of sulfur (S–H at 2368 cm⁻^1^, C–S at 750 cm⁻^1^) and nitrogen (N–H at 3552 cm⁻^1^, C–N at 989 cm⁻^1^) functionalities into the carbon dot structure. Furthermore, Molecular Electrostatic Potential (ESPM) mapping indicated regions of high negative potential around S, OH, and C=O groups, particularly pronounced under acidic and basic conditions, supporting the observed pH sensitivity.

## Introduction

With a current annual production of 66 million tonnes, onions have outpaced all other horticultural crops except tomatoes in terms of global cultivation. The production of onions has risen by more than 25% in the past decade^1,2^. In recent years, the growing demand for processed onions has resulted in a significant increase in waste production^3^. The European Union alone generates over 500,000 tonnes of onion waste annually, primarily from Spain, the United Kingdom, and the Netherlands^4,5^. Onion wastes (OW), consisting primarily of skins, outer scales, roots, and undersized or damaged bulbs, poses a significant environmental challenge. Due to its strong aroma and the rapid proliferation of plant pathogens, OW is unsuitable for use as fodder or organic fertilizer in high concentrations^4^. For that, valorizing wastes such as onion and its extracts could offer a sustainable solution to reduce environmental damage while providing a cost-effective alternative for developing exciting applications. The OW extracts act as sensors due to the color change that change according to pH^6,7^.

At the same time, fluorescent inks (FI) which emit light in the visible or ultraviolet have diverse applications in our daily lives^8,9^. Given their importance, extensive research has focused on developing innovative fluorescent materials to meet growing demands^8,10,11^. The increasing demand for advanced FI has driven research and development efforts to enhance existing materials and explore new nanomaterials with tailored properties^9^. These FIs have diverse applications, including anti-counterfeiting, art and lighting technologies. Security inks for anti-counterfeiting are a particularly valuable application^11–13^. These FIs can be easily and affordably applied to various surfaces and have become widespread in protecting high-value products, documents, and currency. Counterfeiting remains a significant global challenge for businesses, governments, and consumers^9,14^. In addition, art and light technology have embraced fluorescent inks, harnessing their unique properties to create visually striking effects, enhance security features, and illuminate creative expressions. These FIs offer several advantages, including vibrant colors, low cost, ease of application, flexibility for conveying various messages, unique artistic effects, and more^12^. Carbon dots (CDs), a relatively new class of nanomaterials, have emerged as promising candidates for FIs^15–21^. Unlike conventional organic dyes, CDs are composed of carbon-based nanostructures, typically less than 10 nm in size^22–29^. Their unique properties, such as excellent photostability, biocompatibility, and tunable emission spectra, make them attractive for various applications, including FIs^30,31^. Wen et al. developed FIs using green CDs synthesized through pyrolysis and microwave treatment of cotton. These FIs demonstrated promising applications in anti-counterfeiting^32^. Gao et al. developed a new method for synthesizing nitrogen-doped carbon dots (N-CDs) using hydrothermal treatment. The resulting solution exhibited a bright blue fluorescence under UV light and could be used as a FI^33^.

Stimuli-responsive fluorescent materials have garnered significant attention due to their ability to modulate or alter their fluorescent signal in response to external stimuli, such as light, pH, temperature, ions, biomolecules, and solvents^34,35^. These nanoparticles find widespread applications in anti-counterfeiting and data encryption applications. Invisible CDs inks are specifically designed to be undetectable under standard lighting conditions. This invisibility is achieved by carefully engineering the CDs composition to emit fluorescence only when exposed to specific wavelengths of light, such as UV radiation^28,36–38^. A promising approach was predicted to combine inks with other stimuli, like pH, temperature, or ions, to enable reversible switching between multiple colors or fluorescence states^39^. Kim et al. developed innovative dual-responsive nanogels. These nanogels exhibited both photo- and thermo-responsive properties, enabling them to change color in response to light or temperature^40^. Lee et al. created a water-soluble, hyper-branched polymer platform incorporating three stimuli-responsive fluorophores. This platform demonstrated fluorescence changes in response to light, solvent, and cyanide ions^39,41^. While this invisibility can be advantageous for certain applications, such as covert marking or hidden messages, it can also present challenges in terms of detection and verification. If there is no access to UV light or other suitable excitation sources, the invisible ink may be difficult to identify, potentially compromising its effectiveness as a security feature or artistic element. For that, we will use OW as a naked eye pH indicator which will offer a sustainable and cost-effective source for the synthesis of colorimetric pH-sensitive CDs. These CDs can be incorporated into various applications, including security inks, and artistic expressions because of their fluorescence and pH sensitivity.

For security field, pH-sensitive CDs can be incorporated into security features such as labels, packaging, or documents. The color change induced by pH can make it difficult to counterfeit these items, protecting against fraud and counterfeiting^42,43^. In addition, these CDs can be used as authentication markers on valuable items such as artwork, jewelry, or electronics. The unique color change pattern can serve as a reliable means of verifying the authenticity of an object. For artistic decoration and applications, the pH-sensitive CDs can be used to create interactive art pieces that change color or appearance based on the viewer’s touch or the surrounding environment^42^. This dynamic quality can enhance the artistic experience and engage audiences in a new way. While the ink itself is visible, the color change induced by pH can reveal hidden messages or patterns. Artists can use this technique to create intricate works of art that require viewers to decipher the underlying meaning. pH-sensitive CDs can be used to create art that changes over time, reflecting the passage of time or the influence of environmental factors. This can add a layer of depth and complexity to artistic expressions^42,44^.

In this research, we present a novel approach to synthesize a highly pH-responsive and fluorescent ink utilizing sulfur and nitrogen-doped carbon dots (S, N-CDs) derived from readily available onion peel waste through a cost-effective and rapid microwave-assisted method. The novelty of this work lies in the sustainable valorization of onion waste into a multi-functional ink exhibiting reversible fluorescence modulation under varying pH conditions, further elucidated through Density Functional Theory (DFT) calculations and Molecular Electrostatic Potential (MEP) mapping to understand the underlying mechanisms of its pH sensitivity. This unique combination of eco-friendly waste utilization, enhanced pH-responsive fluorescence achieved through S, N-doping, and comprehensive theoretical validation positions our ink as a promising candidate for advanced security features, anti-counterfeiting applications, and innovative artistic expressions, offering a sustainable and versatile alternative to existing materials. We aim to develop a sustainable and efficient method for producing CDs from OW, a readily available and biodegradable waste material. By leveraging the unique properties of CDs, we aim to create novel pH-sensitive inks that can be used in various applications, including artistic expression and security features. This study will not only provide a sustainable solution for waste management but also contribute to the development of innovative materials with potential commercial applications.

## Experimental

### Materials

The onion peel wastes (OW) was obtained from our kitchen, Egypt, and used to prepare S, N-CDs.

### Preparation of carbon quantum dots

To prepare S, N-CDs from OW, a homogeneous solution was prepared by combining 4 gm of OW, 9.33 gm of sodium hydroxide, 9.33 gm of thiourea, and 100 ml of water. After stirring for 30 min, the solution was frozen overnight and then thawed. Ultrasonic treatment was applied for 2 min to disperse any clumps. Finally, the solution was heated in a microwave oven at 700 W for about 7 min.

### Characterization and analysis

Fourier-transform infrared spectra were collected employing Mattson 5000 spectrometer (Unicam, United Kingdom) using the KBr disk method. Fluorescence microscopy was performed using a Jasco FP-6500 spectrofluorometer (made in Japan) with a 150-W xenon arc lamp.

Fluorescence maps were measured over spectra ranges of 300–950 nm (excitation) and 300–400 nm (emission) using Fluorescence spectroscopy was evaluated using the spectrofluorometer model: Jasco FP6500, Tokyo, Japan—light source: Xenon arc lamp 150 Watt.

The UV–vis absorption spectrum was recorded by a UV–Vis spectrophotometer (JASCO V-630, Tokyo, Japan) using a 1 cm path length quartz cell. The quantum yield (QY) was calculated according to the formula:

1 $${\text{QY }} = {\text{ Q}}_{{{\text{st}}.}} \times \frac{mx}{{mst.}} \times \left( {\frac{\eta x}{{\eta st.}}} \right)$$

where “m” is the slope from the plot of fluorescence vs absorbance, the “x” indicates the S, N-CDs, and “st.” refers to methylene blue standard solution in water (0.1 M)^36^.

The Gaussian 09 W program was used to perform density functional theory (DFT) calculations with the hybrid functional B3LYP (Becke’s three-parameter hybrid functional utilizing the BLYP correlation functional) with the 6-31G(d) basis set exhausted by the Berny technique. Different parameters were investigated via DFT calculations, including some of the optimized geometries and ground state energies, including total energy (E_T_), the energy of the highest occupied MO E_HOMO_, the energy of the lowest unoccupied MO E_LUMO_, the energy gap (E_g_), the dipole moment (μ), the absolute electroneg^45^ativity (χ), the chemical density (Pi), the absolute hardness (η), the absolute softness (σ), and the global electrophilicity (ω)^28,45–47^.

2 $$E_{gap} = \left( {E_{LUMO} - E_{HOMO} } \right)$$

3 $${\upchi } = \frac{{{-}{ }\left( {E_{HOMO} + { }E_{LUMO} } \right){ }}}{2}$$

4 $${\text{Pi}} = \, {-}{\upchi }$$

5 $${\upeta } = \frac{{\left( {E_{LUMO} + { }E_{HOMO} } \right){ }}}{2}$$

6 $${\upsigma } = \frac{{1{ }}}{{\upeta }}$$

7 $${\upomega } = \frac{{ Pi^{2} { }}}{{2{\upeta }}}$$

## Results and discussion

### Mechanism of the preparation of S, N-CQDs from onion peels wastes and the pH range response

**Fig. 1:**
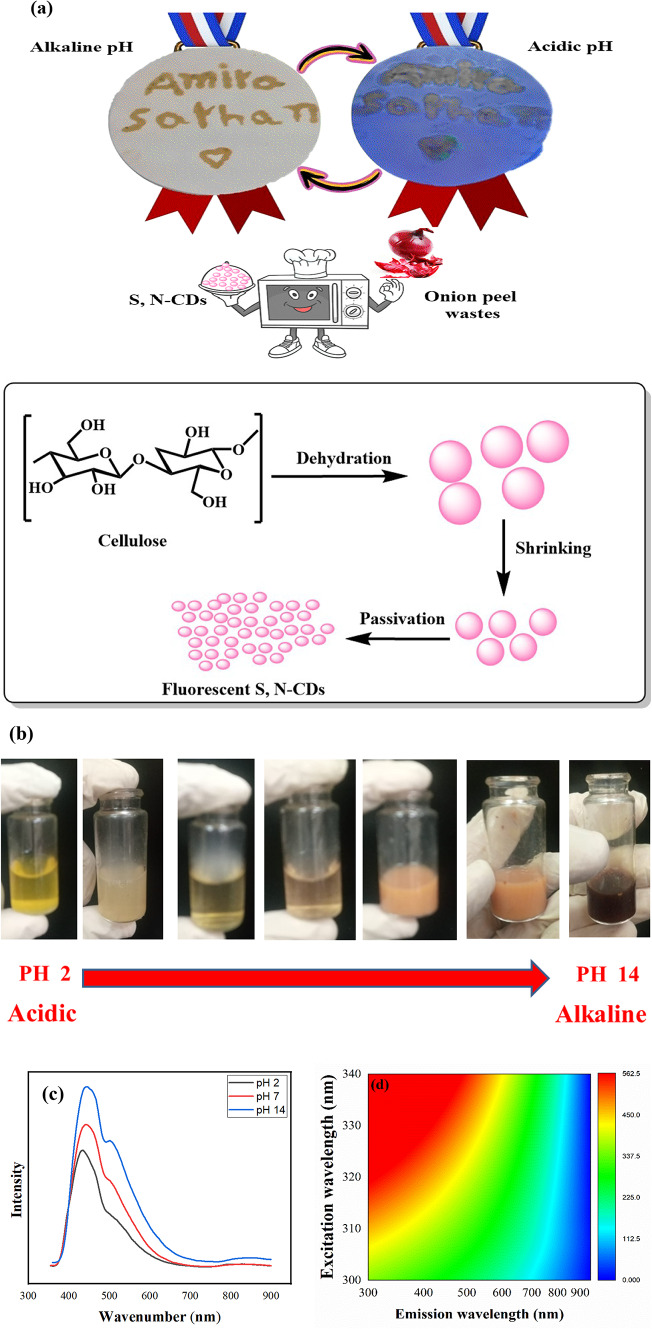

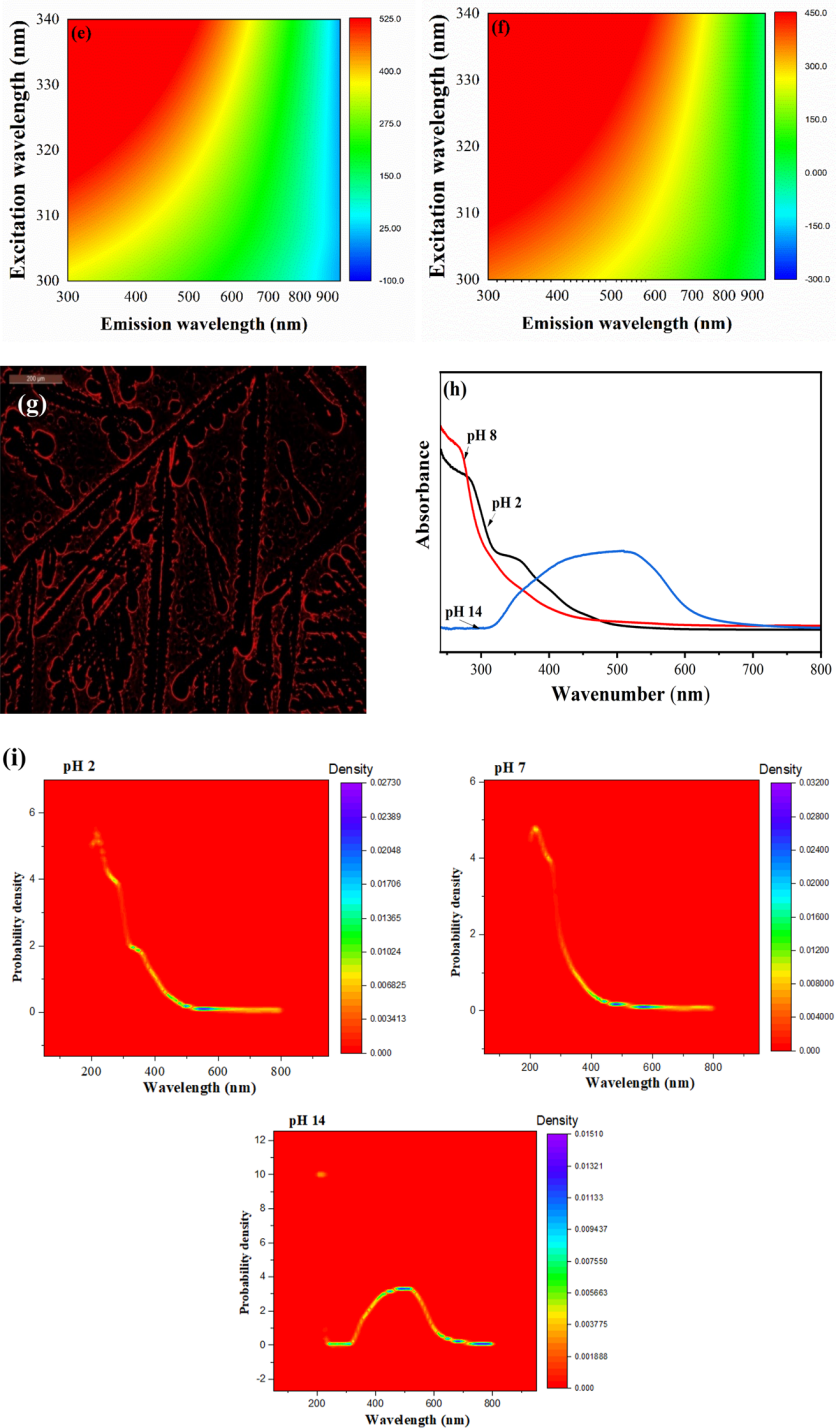
(**a**) Mechanism of the preparation of S, N-CQDs from onion peels wastes, (**b**) pH range response, (**c**) Fluorescent spectra at pH 2, pH 7 and pH 14, (**d**) Contour plots & color mapping at pH 2, (**e**) Contour plots & color mapping at pH 7, (**f**) Contour plots & color mapping at pH 14, (**g**) fluorescence microscope, (**h**) UV spectra of S, N-CDs at different pH values, and (**i**) Kernel plot density of UV at different pH.

The cellulose is the main component of OW^48–50^. Cellulose precursors undergo a transformation during CQDs synthesis. This process begins with dehydration, which breaks down the starting material and leads to the formation of polymers which undergo further dehydration, leading to shrinkage. At the same time, C–C bonds are made, and groups of aromatic atoms start to clump together inside the polymers. When there are enough of these aromatic groups, it starts the process of making CDs^51,52^. In this first stage, functional groups such as OH, C=O, S, N, and COOH groups passivate aromatic clusters that are aggregating on the particle surface (Fig. 1a). These polymer nanoparticles eventually change into CQDs, which results in the ratio of polymer to CDs to drop. As the polymers break down in the final stage, smaller, less polycrystalline S, N-CDs with a narrower size distribution are produced^52^.

The color exhibited by the OW carbon dot extract is highly sensitive to the surrounding pH, primarily due to the presence of flavonoids within the extract^21^. These flavonoid compounds undergo structural transformations in response to varying hydrogen ion concentrations, leading to distinct colorimetric changes. Under acidic conditions, the extract predominantly displays yellow color. This yellow coloration arises from the protonation of the flavonoid molecules. This protonation event disrupts the molecule’s conjugated electron system, influencing how it absorbs light. As the pH of the solution increases towards neutrality, a shift in color towards orange is observed. This transition occurs due to partial deprotonation of the flavonoids. This partial loss of protons leads to an increase in the extent of electron conjugation within the flavonoid structure, consequently altering its light absorption properties. In alkaline environments, the OW carbon dot extract often exhibits a red color. This significant color change is attributed to more substantial structural modifications in the flavonoids. These modifications can include complete deprotonation, as well as potential tautomerization or the formation of quinone and/or phenolate species. These transformations further extend the conjugated system of the flavonoids, resulting in the absorption of light at wavelengths corresponding to red. These vivid color changes across the pH spectrum effectively demonstrate the dynamic interplay between the flavonoid structure, the delocalization of electrons within the molecule, and the resulting light absorption characteristics. While the protonation of the carbonyl oxygen within the flavonoid structure is a key factor influencing these changes in acidic conditions, leading to resonance-stabilized oxonium ions, deprotonation of the carbonyl group in alkaline media to form resonance-stabilized enolate ions also plays a crucial role in the observed red coloration. The specific arrangement of rings and the presence of substituents on the flavonoid skeleton can further modulate this pH-dependent color response (Fig. 1b).

To investigate the optical properties of the prepared ink, Fig. 1c shows the fluorescence emission spectra of the S, N-CDs at different pH. They were stimulated at 350 nm and emitted at wavelengths of 435.00, 443.00 and 444.00 nm due to the C=O/C=N/C=S moieties^53^. An additional peak at 501.00, 502.00 and 503.00 nm is ascribed to oxygen vacancy defects^54,55^. In theory, this emission might demonstrate the potential of these materials as sensors. Figure 1d–f show the fluorescence contour maps (FCM) which showed multicolor emission of S, N-CDs at different pH. The FCM is a unique fingerprint for each material. For that, there is a difference between the three FCMs at pH 2, 7 and 14. In acidic conditions (pH 2), protonation of nitrogen-containing groups alters the electronic environment, creating energy traps or surface states that favor higher-energy blue light emission. Additionally, protonation may modify surface states or enhance quantum confinement, contributing to this blue shift (Fig. 1d). Conversely, in alkaline conditions (pH 14), deprotonation of oxygen- and sulfur-containing groups such as carboxyl, hydroxyl, and thiol groups introduce negative charges and alters electron distribution. This can lead to the formation of new emissive centers or charge-transfer transitions involving lower energy levels, resulting in a red shift and enhanced orange emission (Fig. 1f). The presence of both S and N doping is crucial, with S doping modifying the electronic band structure and introducing emissive traps, and N doping providing pH-sensitive groups that undergo protonation and deprotonation, thus enabling tunable fluorescence behavior. Figure 1g shows the fluorescence microscope of S, N-CDs. The S, N-CDs show a red colored leafy shaped fluorescence. These findings confirmed the efficacy of S, N-CDs synthesized from OW as a suitable material for future use in chemical sensing applications.

The UV–vis spectrum of S, N-CDs in Fig. 1h shows typical optical absorption in the UV region. At normal pH, the spectrum has two main absorption features: an intensive peak at 264.50 nm was assigned to the π–π* of C=C bonds^56,57^. A shoulder peak at 356.50 nm was due to the n–π* transition of C=O bonds^22^. Upon protonation at pH 2, the UV–Vis spectrum exhibited shifts to 274.50 nm and 351.00 nm, indicating oxonium ion formation. The protonation of particular locations on the flavonoid, like hydroxyl groups on the aromatic rings, can expand the molecule’s conjugated system, thereby enhancing electron delocalization. This expanded conjugation reduces the energy difference between electronic states, enabling the absorption of lower energy photons (longer wavelengths) for electronic transitions, which manifests as a bathochromic shift or red shift in the UV absorption peak (as illustrated in Fig. 1h). Conversely, in a highly alkaline environment (pH 14), a shift to 516.00 nm was observed, suggesting the deprotonation of the flavonoid’s phenolic hydroxyl groups, resulting in the formation of phenolate ions (akin to enolate formation). This phenolate formation increases electron density and extends the conjugated system, again causing a red shift. The absence of the C–C peak in the UV–Vis spectrum of flavonoids under alkaline conditions can be explained by substantial changes in the electronic structure resulting from the generation of phenolate ions through the loss of protons from phenolic hydroxyl groups. This deprotonation disrupts the conjugation associated with the C–C bond, leading to alterations in electronic transitions and potentially the disappearance or significant reduction of the absorbance related to this bond^21^. The calculated QY for S, N-CDs was 37.76%.

The differences in the UV–vis kernel density estimation (KDE) plots for S,N-CDs at pH 2 and pH 14 provide valuable insights into the material’s behavior under varying acidity. A KDE plot visualizes the probability density of absorbance values across different wavelengths. A higher peak in the KDE plot indicates a greater likelihood of finding absorbance values at that specific wavelength. When observing a line in the KDE plot at pH 2, it suggests that the absorbance is concentrated within a narrow range of wavelengths. This implies that, under acidic conditions, the S,N-CDs exhibit a strong and consistent absorption at a particular wavelength, with minimal variation. The protonation of specific sites, such as hydroxyl groups, likely contributes to a more homogeneous electronic environment within the nanoparticles, resulting in this consistent absorption pattern. The “increased intensity” of this line further at neutral conditions indicates a high probability density, meaning a large portion of the S,N-CDs display very similar absorption behavior. Conversely, a “bended” line in the KDE plot at pH 14 indicates a broader distribution of absorbance across a wider range of wavelengths. This suggests that the S,N-CDs exhibit more varied absorption behavior under alkaline conditions. The bending of the line signifies that the probability density varies across the spectrum, reflecting variations in absorbance. At this higher pH, the deprotonation of different functional groups leads to a greater variety of electronic states within the S,N-CDs. This increased heterogeneity in electronic transitions results in a broader absorption spectrum, which is captured by the bent shape of the KDE plot (Fig. 1i).

### Molecular electrostatic potential mapping under different pH conditions

**Fig. 2:**
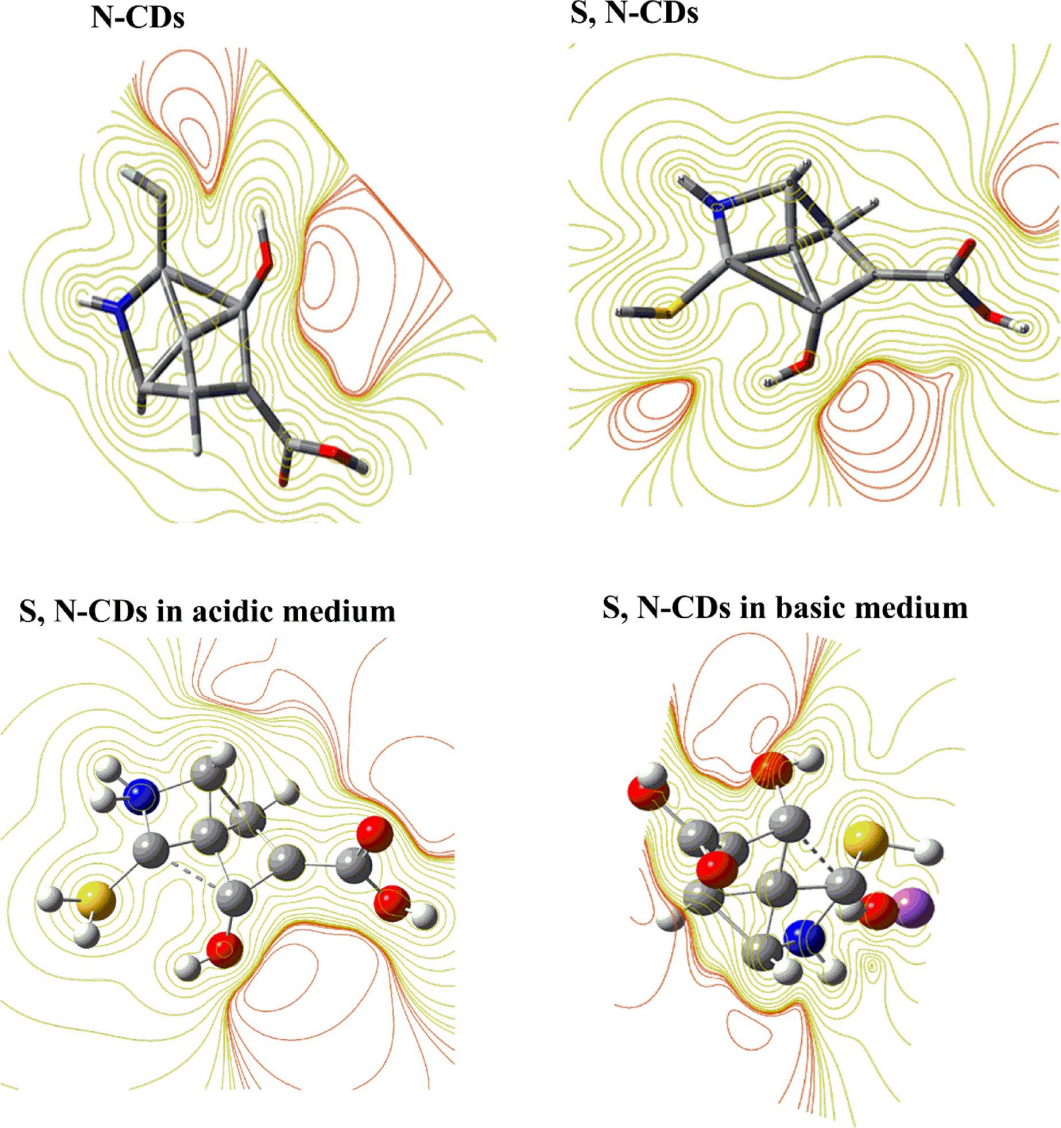
The ESPM for the N-CDs and S, N-CDs at normal pH; and S, N-CDs at acidic and basic medium (red regions indicate negative potentials (nucleophilic-donor coordination sites) and blue regions positive electrostatic potentials (electrophilic-acceptor coordination sites).

The Molecular electrostatic potential map (ESPM) helps identify reactive sites and predict molecular interactions, including electrophilic and nucleophilic attacks as well as hydrogen bonding. It’s crucial for predicting electrophilic and nucleophilic reactions. We used B3LYP/6-31G(d,p) to calculate ESPM for S, N-CDs. The negative electrostatic potential is indicated by red regions, the blue region indicates the positive electrostatic potential, the yellow region reveals the slightly rich electron and the green region shows neutral potential^58^. At neutral conditions, the ESPM for the N-CDs and S, N-CDs was calculated and the result, reported in Fig. 2, evidences, as expected, a high negative electrostatic potential region around S, OH and C = O in the case of S, N-CDs while N-CDs show just negative potential regions around OH and C = O. This high negative electrostatic potential in S, N-CDs is likely due to the electronegativity of these atoms, which causes them to attract protons towards themselves. This can lead to stronger interactions between the S, N-CDs and protons, as its properties can change depending on the pH of the environment which is important for pH-sensitive behavior. The ESPM of S, N-CDs exhibits the most intense red regions, signifying the highest negative electrostatic potential, under both acidic and basic conditions. This finding is further substantiated by the dipole moment values (calculated in DFT), which show the following: basic > acidic > neutral.

### DFT computational calculations under different pH conditions

**Fig. 3:**
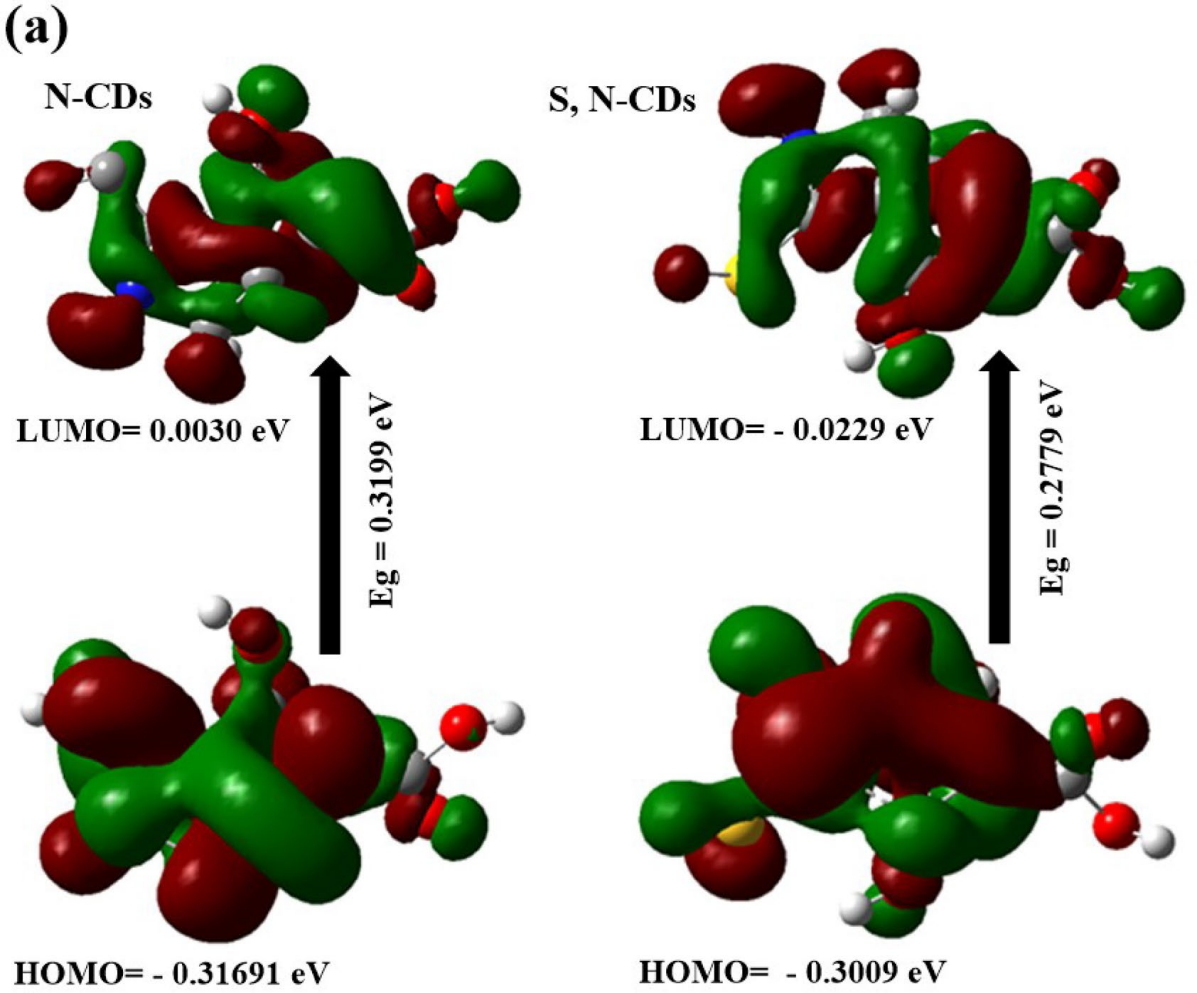

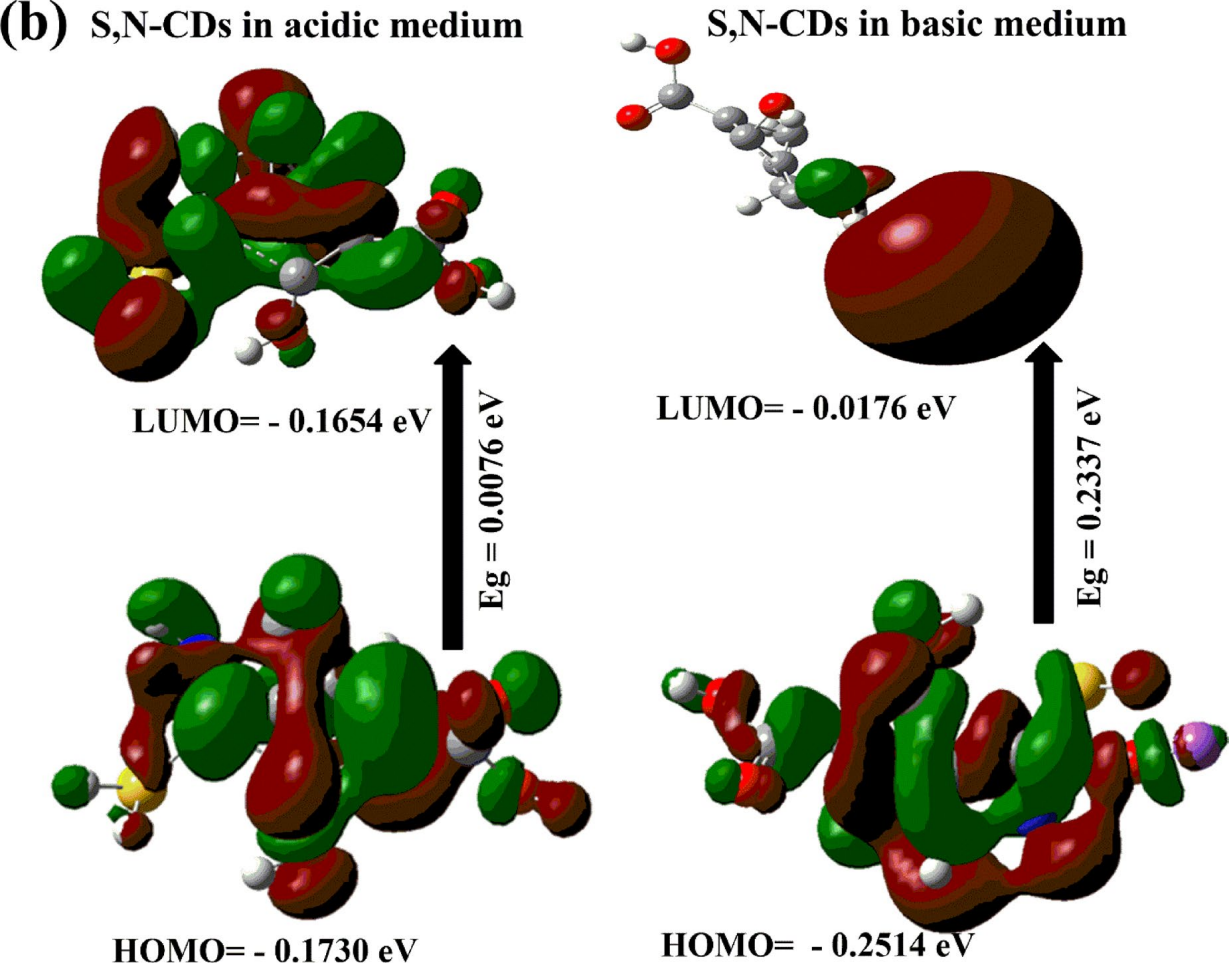
Frontier molecular orbitals of were calculated using DFT B3LYP/6–31G for (**a**) N-CDs and S, N-CDs at neutral medium; and (**b**) S, N-CDs under acidic and basic medium.

To investigate the potential of S, N-doped carbon dots (S, N-CDs) as pH-sensitive inks compared to N-CDs, we synthesized both N-only and S,N-doped CDs. DFT calculations were then performed to assess their electronic structures. The results revealed that S, N-doped CDs exhibited a more favorable electronic structure for pH-induced color changes. The presence of S atoms enhanced the S,N-CD’s reactivity towards protons, leading to more pronounced shifts in absorption spectra and visible color variations under different pH conditions. This suggests that S, N-CDs are promising candidates for developing highly sensitive and responsive pH-sensitive inks and this challenged us to prepare S,N-CDs instead of N-CDs. From Fig. 3 and Table 1, the results show the following:

**Table 1:** The quantum chemical parameters of N-CDs and S, N-CDs at normal pH and S,N-CDs at different pH values.

DFT B3LYP/6–31G (d)	N-CDs	S, N-CDs		
	Neutral	Acidic	Neutral	Basic
E_LUMO_ (eV)	0.0030	− 0.1654	− 0.0229	− 0.0176
E_HOMO_ (eV)	− 0.3169	− 0.1730	− 0.3009	− 0.2514
E_T_ (au)	− 581.27	− 944.07	− 939.24	− 1174.19
E_g_ (eV)	0.3199	0.0076	0.2779	0.2337
μ (Debye)	1.761	6.67	2.640	12.45
χ (eV)	0.1569	0.1692	0.1619	0.1345
Pi (eV)	− 0.1569	– 0.1692	− 0.1619	− 0.1345
σ (eV)	− 6.3716	− 5.9094	− 6.1760	− 7.4313
ω (ev)	− 0.0784	− 0.0846	− 0.0809	− 0.0672

- The E_g_ for S,N-CDs (i.e., 0.2779 eV) compared to N-CDs (i.e. 0.3199 eV), confirming the S, N-CDs reactivity in comparison with the N-CQDs. A narrower E_g_ can make S,N-CDs more sensitive to changes in its electronic structure, which is essential for creating pH-sensitive inks that respond visually to changes in pH.
  In addition, the Pi of S,N-CDs (i.e. − 0.1619) is more negative compared to N-CDs (i.e. − 0.1569 eV), which means the higher reactivity of S, N-CDs, which can increase its sensitivity to changes in pH^59^. This is also confirmed from the high μ for S, N-CDs (i.e. 2.640 Debye). The high μ creates a polar environment within the S, N-CDs, which can facilitate interactions with protons (H^+^ ions) present in acidic or basic solutions.
- The S, N-CDs are more electrophilic (− 0.0809 eV), refers to their strong tendency to attract electrons, compared to the N-CQDs (− 0.0784 eV) because of the presence of S and N. An increase in the ω value for S,N-CDs is a good approximation of strong energy changes between the donor (HOMO) and acceptor (LUMO)^60^. When exposed to acidic conditions, these S, N-CDs can easily accept protons, leading to a change in the electronic structure of the S, N-CDs. These changes in optical properties can result in visible color shifts, making the S, N-CDs a pH-sensitive ink.
- A less stable S, N-CD (i.e. E_T_ =  − 939.24 au) may be more reactive due to its higher energy state. This can be beneficial for applications that require quick responses such as pH-sensitive ink (Fig. 3a).

To study the effect of pH on the DFT calculations and geometry we find that, the observed narrower E_g_ for S, N-CDs in acidic medium (0.0076 eV) compared to both neutral (0.2779 eV) and basic conditions (0.2337 eV), coupled with a higher dipole moment in the acidic case (6.67 Debye), likely arises from the specific alterations in the electronic structure and charge distribution induced by protonation. In an acidic environment, the surface of the S, N-CDs becomes enriched with protons, leading to the protonation of various nitrogen and oxygen-containing functional groups such as amines, pyridinic nitrogen, carboxyl, and hydroxyl groups. This protonation can introduce new electronic states within the band gap or modify existing ones, effectively reducing the energy difference between the HOMO and LUMO. Furthermore, the addition of positive charges through protonation, particularly if it occurs unevenly across the nanoparticle surface, can lead to a greater separation of charge centers and an alignment of the dipole moments of the newly formed ionic species. This asymmetry in charge distribution, along with enhanced interactions with the polar water solvent in acidic conditions, contributes to the observed higher overall dipole moment. Conversely, in a basic medium, the deprotonation of acidic functional groups like carboxylic acids and phenols results in a negatively charged surface. This different set of surface modifications and electronic states associated with the deprotonated species can lead to a wider E_g_ compared to the protonated state. The distribution of negative charges upon deprotonation might also result in a less polar overall structure or a different alignment of surface dipoles, explaining the lower dipole moment compared to the acidic environment. Therefore, the unique chemical transformations occurring on the S, N-CDs surface under different pH conditions directly influence their electronic transitions and charge distribution, ultimately dictating the observed changes in the energy gap and dipole moment.

The total energy from DFT calculations was found to follow the following order: basic < acidic < neutral. This suggests that the most stable S, N-CD configuration is produced by the basic medium, which is followed by the acidic medium, while the least stable form (highest total energy) is produced by the neutral medium (Fig. 3b). Negatively charged carboxylate ions are created when acidic functional groups on the surface of S, N-CDs, such as carboxylic acids and phenols, deprotonate in a basic medium. Stronger intramolecular or intermolecular interactions may be facilitated by these deprotonated forms (e.g., electrostatic attractions, hydrogen bonding networks involving the newly formed negative charges and surrounding solvent molecules or other parts of the CD structure). The basic form is the most stable because of these stabilizing interactions, which can reduce the system’s total energy. Furthermore, a more energetically advantageous overall electronic structure for the carbon dot may result from the particular arrangement and electronic distribution brought about by deprotonation.

In contrast, the protonation of basic functional groups such as amines results in the introduction of positive charges onto the surface of S, N-CDs in an acidic medium. The presence of several positive charges may also introduce some destabilizing electrostatic repulsions within the nanoparticle, even though these protonated forms can improve interaction with the polar solvent. The protonated species’ unique electronic structure and the balance between advantageous solvation and possibly unfavorable intramolecular repulsions probably lead to an intermediate total energy, which makes it more stable than the neutral but less stable than the basic form.

On the other hand, the neutral S, N-CDs may show fewer strong stabilizing interactions because they lack the substantial surface charges brought on by strongly acidic or basic conditions. In the neutral state, the dipole–dipole interactions of the intrinsic polar functional groups, the balance of van der Waals forces, and interactions with the solvent may not be as energetically advantageous as the states with more noticeable ionic character brought about by protonation or deprotonation. As a result, of the three pH levels, the neutral form has the highest total energy and, consequently, the lowest relative stability.

### FTIR spectroscopy

**Fig. 4:**
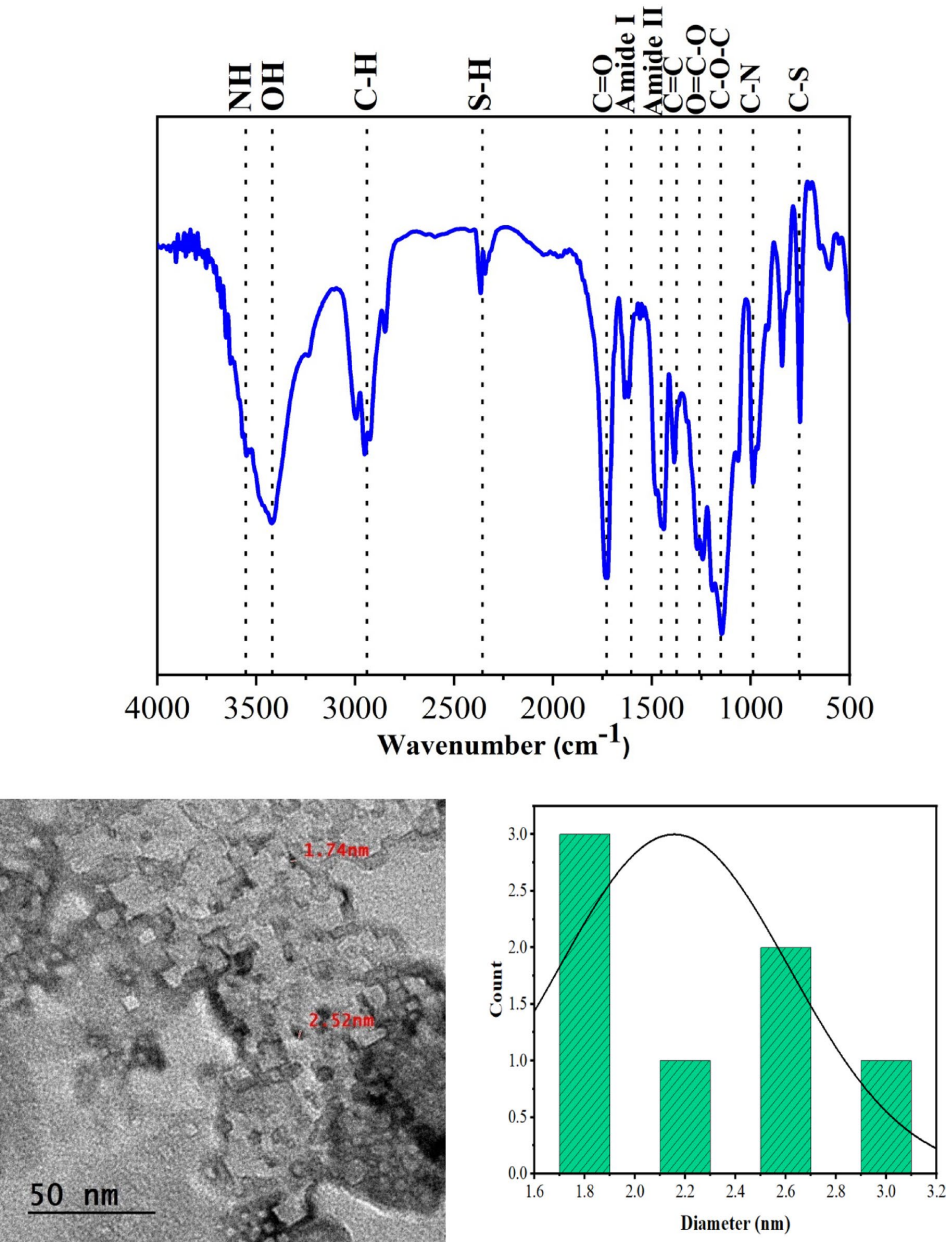
(**a**) FTIR spectra and (**b**) TEM analysis of S, N-CDs with particle size distribution.

To prove the S, N doping we used FTIR spectroscopy. Figure 4a displayed the FTIR spectra of the prepared S, N-CDs displayed absorption peaks between 3423 cm^−1^ (O–H), 2954 cm^−1^ (C–H), 1733 cm^−1^ (C=O), 1384 cm^–1^ (C=C), 1247 cm^−1^ (O–C=O) and 1139 cm^−1^ (C–O–C)^29,54,61^. The peaks at 3552 cm^-1^ (N–H), 1617 cm^−1^ (amide I), 1440 cm^−1^ (amide II) and 989 cm^−1^ (C–N) for S, N-CQDs prove the N-doping of CDs^46,62^. While the peaks at 2368 cm^−1^ (S–H), and 750 cm^−1^ (C–S) for S, N-CQDs prove the S-doping of CDs^56,63^. The relative absorbance for C–N and C–S band was used to quantify the degree of substitution (DS) for N and S, respectively. The DS was 0.96 and 1.03 for N and S, respectively.

### Transmission electron microscopy (TEM) analysis

To prove its nano scale within CDs range we made TEM analysis. Figure 4b shows the typical TEM image of CDs. The S, N-CDs exhibited nano-doted spherical dots with diameter less than 10 nm which proved the preparation of CDs.

### The pH-sensitive S, N-CDs ink

**Fig. 5:**
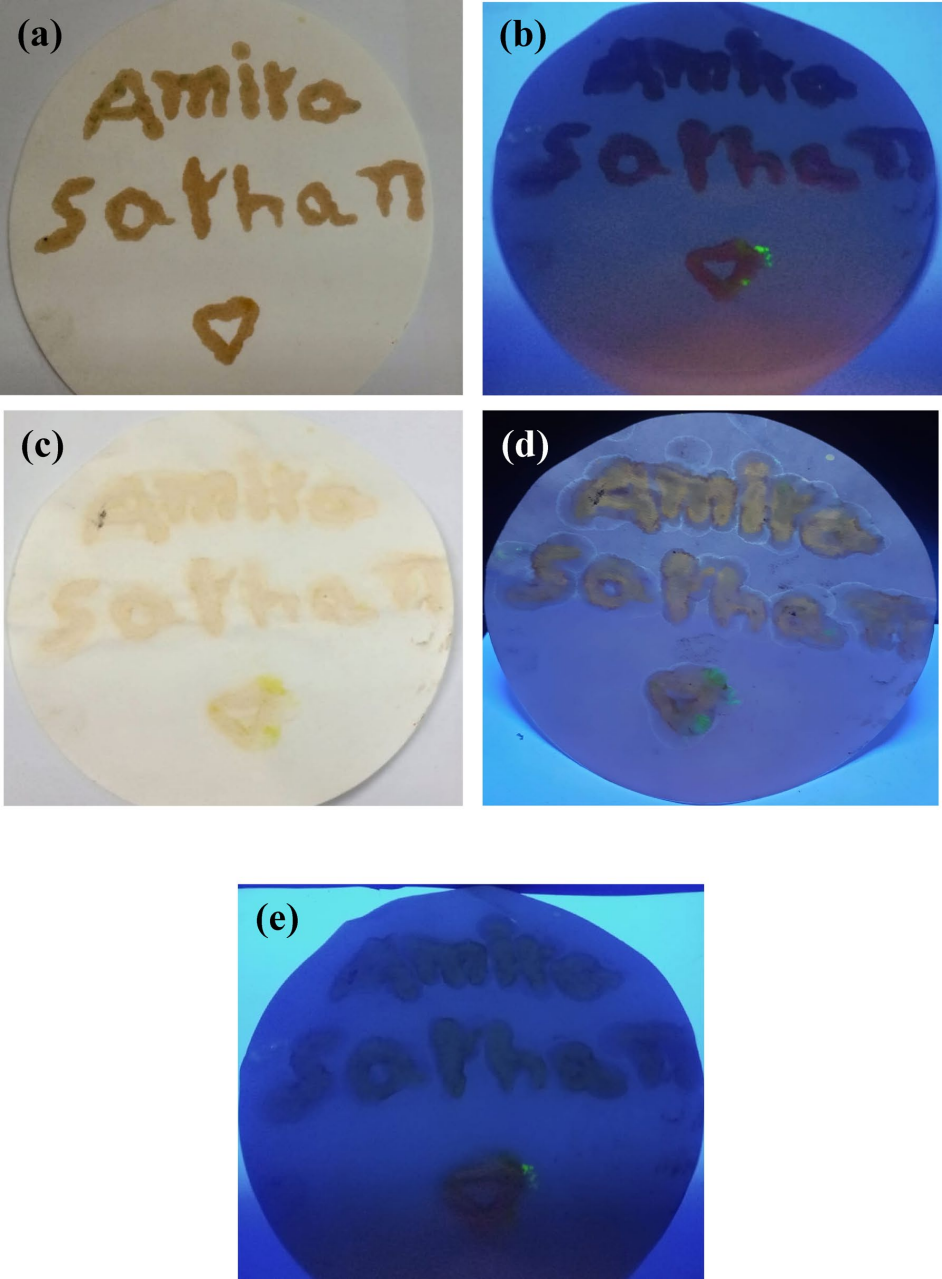
The pH-sensitivity test for the prepare S, N-CDs ink (**a**) Once the words wrote by S, N-CDs under light, (**b**) Once the words wrote by S, N-CDs under UV lamp, (**c**) After treatment with acid under light, (**d**) After treatment with acid under UV lamp, and (**e**) After treatment with alkaline under UV lamp.

The observed fluorescence behavior of the S, N-CDs ink demonstrates its pH-sensitive nature. The initial lack of fluorescence under UV light suggests that the S, N-CDs in the ink are in a non-fluorescent state. However, upon treatment with acid, the ink exhibits a faint yellow color under light and fluoresces under UV light. This indicates a shift in the electronic structure of the S, N-CDs, likely due to protonation. The return to non-fluorescence after re-treatment with alkaline solution suggests that the de-protonation process reverses the effect of acid, restoring the S, N-CDs to their original non-fluorescent state. This reversible pH-sensitivity is a valuable property for applications such as chemical sensing, artistic, or anti-counterfeiting technologies (Fig. 5).

The pH-sensitivity of the S, N-CDs ink offers potential applications in various fields, including: security features applications because of the reversible fluorescence change which can be used to test the originality of the brands covert markings or hidden messages that are only visible under specific conditions. The ink can be incorporated into labels or tags that change color or fluorescence intensity when exposed to certain pH values. This can serve as a reliable authentication mechanism for valuable items. In addition, in the artistic applications because of the ink can be used to create interactive art pieces that change color or appearance based on pH, providing a dynamic and engaging experience. Moreover, in the chemical sensing where the fluorescence intensity can be used as a quantitative indicator of pH, enabling the development of simple and portable pH sensors. The reversible change in fluorescence intensity under acidic and alkaline conditions can be exploited to create unique and difficult-to-replicate security features.

## Conclusions

The study successfully demonstrates the development of a pH-sensitive fluorescent ink based on S, N-doped CDs synthesized from onion peels. The ink exhibits a reversible change in fluorescence intensity under acidic and alkaline conditions, making it a promising candidate for various applications. Our DFT calculations helped us to decide which CDs we will prepare. From the DFT calculations we decided to prepare S, N-CDs instead of N-CDs because of the high reactivity of S, N-CDs which will be more flexible and will change their color easily with the pH. The FTIR proved the incorporation of S and N atoms inside the S, N-CDs while TEM proved the preparation of S, N-CDs in nano-range. The calculated electrostatic maps proved the flexibility of S, N-CDs to accept protons from the acidic solution. After that, we applied the S, N-CDs as an ink and we saw that the change in color depending on the pH value. In summary, this work effectively illustrates the creation of a pH-sensitive fluorescent ink based on S, N-doped CDs made from easily accessible onion peels. The ink is a promising option for a number of uses, such as security features, anti-counterfeiting measures, and creative artistic expressions, because it shows a reversible change in fluorescence intensity under acidic and alkaline conditions. The enhanced reactivity of S, N-CDs towards protons, which underlies their pH-responsive behavior, was clarified by our DFT calculations. Additionally, the improved proton interaction capabilities of the S, N-CDs were supported by electrostatic potential mapping. The ink’s visible and fluorescent reaction to pH changes demonstrated its practical applicability. This study paves the way for the creation of multipurpose, sustainable inks made from various bio-wastes in the future. The stability of the ink could be improved, its colorimetric and fluorescence range could be increased, and its incorporation into smart materials and devices for enhanced security and creative uses could be investigated further. A new generation of ecologically friendly and technologically advanced materials could be made possible by applying the concepts shown here to the design of responsive inks for other stimuli.

## Data Availability

Data is provided within the manuscript.
